# Enhancing the quality and oxidative stability of sunflower oil during thermal treatment with an ultrasound-extracted pomegranate flower (*Punica granatum* L.) extract

**DOI:** 10.3389/fnut.2026.1797519

**Published:** 2026-03-06

**Authors:** Rehab F. M. Ali, Raed Alayouni, Ayman M. El-Anany

**Affiliations:** 1 Department of Food Science and Human Nutrition, College of Agriculture and Food, Qassim University, Buraydah, Saudi Arabia; 2 Special Food and Nutrition Department, Agricultural Research Center, Food Technology Research Institute, Giza, Egypt

**Keywords:** antioxidants, natural preservatives, oxidative stability, pomegranate flower, quality, sunflower oil, thermal degradation, ultrasound assisted extraction

## Abstract

**Introduction:**

This study investigated the effect of ultrasound assisted pomegranate flower (*Punica granatum L*.) extract (DPF) on the oxidative stability of sunflower oil during intermittent heating. Dried pomegranate flowers were extracted using 80% aqueous ethanol in an ultrasonic bath, yielding a phytochemically rich extract characterized by high levels of total phenolics (267.36 ± 4.19 mg GAE/g DW), flavonoids (84.56 ± 2.43 mg QE/g DW), and ellagitannins such as punicalagins and ellagic acid. The extract exhibited strong *in vitro* antioxidant activity in the DPPH radical scavenging assay.

**Methods:**

Sunflower oil was fortified with DPF extract at concentrations of 400, 800, 1,600, and 2,400 μg/g oil, with 200 μg/g BHT as a synthetic reference. All samples were heated at 180 ± 5°C for 2 h daily over 5 consecutive days. The oil's oxidative stability was assessed by monitoring physicochemical indices including refractive index, viscosity, acid value, peroxide value, iodine value, total polar compounds (PCC), polymer content (PC), and thiobarbituric acid (TBA) value.

**Results and discussion:**

Results demonstrated that DPF incorporation significantly enhanced the oil's thermal oxidative stability in a dose dependent manner. Higher concentrations (1,600 and 2,400 μg/g oil) provided superior protection across all parameters, often outperforming 200 μg/g oil BHT. The oil enriched with 2,400 ppm DPF proved most effective, it best preserved the iodine value while mitigating increases in refractive index, viscosity, acid value, peroxide value, PCC, PC, and TBA value. The findings indicate that ultrasound extracted pomegranate flower antioxidants, particularly at 2,400 μg/g oil, can serve as a potent natural alternative to synthetic antioxidants, effectively prolonging the frying life and maintaining the quality of sunflower oil under repeated thermal stress.

## Introduction

Vegetable oils are essential components of the human diet, serving as a primary source of dietary fats. However, their high concentration of unsaturated fatty acids makes them susceptible to oxidation, a process accelerated by environmental factors such as temperature, light, and metals (1). This degradation leads to the formation of harmful compounds, reduces nutritional quality, and causes undesirable sensory changes like off-flavors (2). Sunflower oil exemplifies this vulnerability, undergoing significant oxidative damage at high cooking temperatures. Its stability under heat depends on factors such as fatty acid composition, heating duration, and the presence of antioxidants. While relatively stable at lower temperatures (150–180°C), it experiences pronounced degradation at higher ranges (210–240°C), characterized by the breakdown of polyunsaturated fatty acids and an increase in saturated and trans fats (3).

To counteract this, antioxidants are often added to enhance the oxidative stability of oils (2). Natural extracts are particularly promising. For example, rosemary and marjoram extracts have been shown to reduce oxidation markers during repeated heating, sometimes outperforming synthetic antioxidants like BHA (4). Similarly, pandan leaf extract stabilizes oil during microwave heating by lowering peroxide and *p*-anisidine values (5), while oleoresins from thyme and rosemary help maintain oil quality and reduce harmful compound formation during prolonged heating (6). Given the health risks associated with oxidation byproducts, including potential links to cardiovascular disease, such stabilization methods are crucial (1).

Among potent natural sources, pomegranate flower extract is notable for its high phenolic (14.82–90.86 mg GAE/g) and flavonoid (up to 500 mg QUE/g) content (7, 8). Its efficacy is highly dependent on the extraction solvent, with methanol yielding the most active extracts for antioxidant activities like DPPH radical scavenging (8, 9). These extracts have demonstrated superior antioxidant capacity compared to synthetic standards such as BHT and α-tocopherol (7). Beyond oil stabilization, their bioactive compounds are associated with various health benefits, including anti-inflammatory, hepatoprotective, anti-cancer, and cardiovascular protective effects (10–12), as well as antibacterial activity (9).

To fully harness this potential, efficient extraction techniques are vital. The efficiency of extracting bioactive compounds from plant materials is a critical factor determining the ultimate antioxidant efficacy in oils. Among modern extraction techniques, ultrasound-assisted extraction (UAE) has emerged as a highly effective and environmentally friendly method. UAE operates on the principle of acoustic cavitation, where ultrasonic waves generate and implode micro-bubbles in the solvent, leading to cell wall disruption and enhanced mass transfer (13, 14). This process significantly increases the yield of extracted phenolic compounds and reduces extraction time and solvent consumption compared to conventional methods such as maceration and Soxhlet extraction (15). Specifically, UAE can achieve extraction in 146 min compared to 6–24 h for Soxhlet, while operating at moderate temperatures (72°C) that preserve thermosensitive bioactive compounds like ellagitannins and flavonoids, which are often degraded during prolonged Soxhlet extraction (15). For oil fortification, UAE offers the additional advantage of facilitating the direct incorporation of antioxidants into the oil matrix without the need for prior extract concentration or the use of organic solvents, aligning with green chemistry principles and “clean-label” trends (13). Recent studies have successfully applied UAE to enrich vegetable oils with antioxidants from various botanical sources, demonstrating improved oxidative stability. For instance, Ahmed et al. (13) showed that direct ultrasonic enrichment of sunflower oil with saffron by-products resulted in significantly enhanced resistance to oxidation during accelerated storage. This integrated approach combining potent natural extracts with advanced extraction technology offers a sustainable strategy to enhance oil quality.

Therefore, this study aims to investigate the oxidative stability of sunflower oil fortified with ultrasound-assisted pomegranate flower extract at concentrations of 400, 800, 1,600, and 2,400 μg/g oil, using 200 μg/g oil of butylated hydroxytoluene (BHT) as a synthetic reference, during intermittent thermal exposure at 180 ± 5°C.

## Materials and methods

### Plant material

Dried Pomegranate Flowers (DPF, 5A Grade) were sourced from Bozhou, Anhui, China. The material, marketed under the brand Xanadu, consisted of 100% pure, dried red flower pieces. It was stored in a cool, dry place and used within its 12-month shelf life for extraction.

The dried pomegranate flowers were ground using an electric laboratory mill (MF 10 basic, IKA-Werke, Germany) operated at 3,000 rpm in 30–60 s intervals to prevent overheating. The ground material was then sieved through a series of standard test sieves (Endecotts, London, UK) mounted on a mechanical sieve shaker (Retsch AS 200, Germany) for 10 min at amplitude 1.5 mm. The fraction passing through a 1.0 mm mesh sieve but retained on a 0.5 mm mesh sieve (particle size range: 0.5–1.0 mm) was collected for extraction. This particle size range was selected to optimize the balance between surface area availability and practical handling during UAE, while avoiding the filtration difficulties associated with excessively fine powders. The collected fraction was thoroughly homogenized by manual mixing in a polyethylene bag for 5 min. Homogeneity was verified by determining the coefficient of variation for extract yield and total phenolic content from five random samples, which was less than 6% for both parameters. The homogenized powder was stored in airtight containers at room temperature until extraction.

### Chemicals and reagents

All chemicals and reagents used were of analytical grade and supplied by Sigma Chemical Co. (St. Louis, MO, USA).

### Sunflower oil

Refined, antioxidant free sunflower oil (NDC-UAE, supplied by KSA Royal Star LLC, Oman) was used, with initial peroxide and acid values of 2 mEq O_2_/kg and 0.05 mg KOH/g oil, respectively.

### Ultrasound- extraction of DPF

An ultrasound-assisted extraction (UAE) protocol was employed to obtain the DPF extract. Briefly, 100 g of DPF powder was mixed with 620 ml of 80% aqueous ethanol (v/v 496:124) and sonicated in an ultrasonic bath (20 kHz) at 72°C for 146 min. The mixture was subsequently centrifuged at 3,000 rpm for 10 min, and the extraction process was repeated twice on the residue. The combined supernatants were filtered through Whatman No. 1 filter paper. The filtrate was concentrated under reduced pressure using a rotary evaporator at 45°C, and the resulting crude extract was stored at −18°C until further use (16).

The extraction yield was calculated as the percentage ratio of the weight of dried crude extract obtained to the initial weight of dried pomegranate flower powder used. The yield was found to be 21.25%. For phytochemical analysis, aliquots of the concentrated extract were completely dried in a vacuum oven at 40°C until constant weight was achieved.

The extraction parameters were selected based on preliminary single-factor experiments and literature review to optimize the recovery of phenolic compounds. The solvent concentration of 80% aqueous ethanol was chosen to provide a balance between polarity and extraction efficiency for both phenolic acids and ellagitannins. The temperature of 72°C was selected to enhance solubility and mass transfer while minimizing thermal degradation of thermosensitive compounds. The extraction time of 146 min was optimized to ensure exhaustive recovery of cell-bound phenolics using the indirect ultrasonic bath system. The solid-to-solvent ratio of 1:6.2 (w/v) was selected to maintain an adequate concentration gradient while minimizing solvent consumption for subsequent concentration steps.

### Phytochemical analysis of DPF

The DPF extract was subjected to comprehensive phytochemical analysis. Total phenolic content was determined using the Folin-Ciocalteu colorimetric method following the procedure described by Singleton et al. (17), with gallic acid as the reference standard. Briefly, an aliquot (0.5 ml) of the ethanolic extract was mixed with 5 ml of freshly prepared 0.2 N Folin-Ciocalteu reagent. After 5 min of incubation, 4 ml of sodium carbonate solution (75 g/L) was added, and the mixture was allowed to stand in the dark at 25°C for 120 min. The absorbance was then measured at 765 nm using a UV-Vis spectrophotometer (UNICO 2100A, USA). A calibration curve was constructed using gallic acid standards, and the results were expressed as milligrams of gallic acid equivalent per gram of dry weight (mg GAE/g DW). Total flavonoid content was determined following the method described by Zhishen et al. (18), using quercetin as the reference standard. Briefly, a 5 ml aliquot of the sample extract was mixed with 5 ml of a 2% aluminum chloride (AlCl_3_) solution prepared in methanol. The mixture was incubated at room temperature for 10 min, after which the absorbance was measured at 415 nm using a UV-Vis spectrophotometer. A calibration curve was constructed using quercetin standards, and the results were expressed as milligrams of quercetin equivalents, QE per gram of dry weight (mg quercetin/g DW). Condensed tannin content (mg Catechin Equivalents (CE)/g DW) was determined following the modified vanillin assay described by Naczk et al. (19). Total anthocyanin content was determined following the method described by Alshammai et al. (20), with results expressed as mg cyanidin-3-glucoside equivalents/g DW. The absorbance was measured at 530 nm using a UV-Vis spectrophotometer. Antioxidant activity was evaluated using the DPPH radical scavenging assay (16), wherein extract solutions (50, 100, 200, and 400 μg/ml) were reacted with a methanolic DPPH solution, and absorbance was measured at 517 nm. The phenolic profile of DPF extract was characterized using an Agilent Technologies HPLC system equipped with a diode array detector (DAD). Separation was achieved on a C18 column (250 mm × 4.6 mm, 5 μm particle size) maintained at ambient temperature. The mobile phase consisted of a gradient elution with solvent A (water/acetic acid) and solvent B (methanol/acetonitrile) at a flow rate of 1.0 ml/min. The injection volume was 50.0 μl. Detection was monitored at three wavelengths: 280 nm for phenolic acids and flavan-3-ols, 320 nm for hydroxycinnamic acids and flavonoids, and 360 nm for flavonols and anthocyanins. Data acquisition and processing were performed using ChemStation software. Phenolic compounds were identified by comparing their retention times and UV-Vis spectra with authentic standards, and quantification was carried out using calibration curves of corresponding standards (8).

### Sample preparation and oil heating protocol

#### Sample preparation

Refined, antioxidant-free sunflower oil (initial peroxide value: 2 mEq O_2_/kg; acid value: 0.05 mg KOH/g oil) was used as the base oil. The ultrasound-extracted DPF was incorporated into the oil at final concentrations of 400, 800, 1,600, and 2,400 μg/g oil. For each concentration, the appropriate amount of DPF extract was weighed and directly added to 750 g of sunflower oil. The mixture was stirred using a magnetic stirrer at 500 rpm for 30 min at ambient temperature (25 ± 2°C) to ensure complete homogenization of the extract within the oil matrix. A comparative sample containing 200 μg/g oil of the synthetic antioxidant butylated hydroxytoluene (BHT) was prepared following the same procedure. A control sample (0 μg/g oil) consisting of pure sunflower oil without any antioxidant addition was also included.

#### Heating protocol

All samples (control, BHT-fortified, and DPF-fortified oils) were subjected to intermittent heating in 26 cm stainless-steel frying pans (Korkmaz A1023 Alfa). The pans were placed on a flat gas stove and heated at 180 ± 5°C for 2 h per day over five consecutive days. During the heating process, the pans remained open and exposed to air, simulating actual frying conditions. To ensure uniform heating and aeration, the oil was manually stirred every hour using a stainless-steel spatula. Between heating cycles, the oil samples were allowed to cool to ambient temperature, and the pans were covered with aluminum foil to protect the oil from light exposure during storage at 5°C. Daily aliquots (approximately 50 ml) were collected in amber glass bottles, flushed with nitrogen, and stored at 5°C in the dark until subsequent analysis.

### Analytical methods for oil quality assessment

The quality of the oil was evaluated through a series of physicochemical and oxidative stability assays as described below. All analyses were performed in triplicate, and results were expressed as mean ± standard deviation.

#### Acid value (AV)

The acid value, representing the free fatty acid content, was determined according to AOAC (21) method Cd 3d-63. Briefly, 10 g of oil sample was accurately weighed into a 250 ml Erlenmeyer flask and dissolved in 50 ml of a neutralized solvent mixture consisting of diethyl ether and ethanol (1:1, v/v). Three drops of phenolphthalein indicator (1% in ethanol) were added, and the solution was titrated with 0.1 N potassium hydroxide (KOH) solution until a persistent pink color appeared for at least 30 s. The acid value was calculated using the following formula and expressed as mg KOH/g oil:


(1) $$\begin{array}{l} \text{AV }\left(\text{mg KOH}/\text{g}\right)=\text{V}\times \text{N}\times 56.1/\text{W } \end{array}$$

where V = volume of KOH used (ml), N = normality of KOH solution (0.1 N), 56.1 = molecular weight of KOH, and W = weight of oil sample (g).

#### Peroxide value (PV)

The peroxide value, measuring primary oxidation products (hydroperoxides), was determined according to AOAC (21) method Cd 8-53. Approximately 5 g of oil sample was accurately weighed into a 250 ml glass-stoppered flask and dissolved in 30 ml of a glacial acetic acid-chloroform solution (3:2, v/v). Saturated potassium iodide solution (0.5 ml) was added, and the flask was immediately stoppered, shaken gently for 1 min, and placed in the dark for exactly 5 min. After incubation, 30 ml of distilled water was added, and the mixture was titrated with 0.01 N sodium thiosulfate (Na_2_S_2_O_3_) solution using 1% starch solution as indicator (added near the endpoint when the yellow color became faint). The titration continued until the blue color completely disappeared. A blank determination was performed simultaneously under the same conditions. The peroxide value was calculated using the following formula and expressed as milliequivalents of active oxygen per kilogram of oil (mEq O_2_/kg):


(2) $$\begin{array}{l} \text{PV }\left(\text{mEq }O2/\text{kg}\right)=\left(\text{S}-\text{B}\right)\times \text{N}\times 1,000/\text{W} \end{array}$$

where S = volume of Na_2_S_2_O_3_ used for sample (ml), B = volume of Na_2_S_2_O_3_ used for blank (ml), N = normality of Na_2_S_2_O_3_ solution (0.01 N), and W = weight of oil sample (g).

#### Iodine value (IV)

The iodine value, measuring the degree of unsaturation, was determined according to AOAC (21) method Cd 1d-92 using the Wij's method. Approximately 0.5 g of oil sample was accurately weighed into a 500 ml glass-stoppered flask and dissolved in 15 ml of chloroform. Wij's solution (25 ml) containing iodine monochloride in glacial acetic acid was added, and the flask was stoppered, shaken gently, and stored in the dark for exactly 30 min. After incubation, 20 ml of 15% potassium iodide solution and 100 ml of distilled water were added. The liberated iodine was titrated with 0.1 N sodium thiosulfate solution using 1% starch solution as indicator (added near the endpoint). A blank determination was performed simultaneously. The iodine value was calculated using the following formula and expressed as grams of iodine absorbed per 100 g of oil (g I_2_/100 g oil):


(3) $$\begin{array}{l} \text{IV }\left(\text{g }{\text{I}}_{2}/100\text{ g}\right)=\left(\text{B}-\text{S}\right)\times \text{N}\times 12.69/\text{W} \end{array}$$

where B = volume of Na_2_S_2_O_3_ used for blank (ml), S = volume of Na_2_S_2_O_3_ used for sample (ml), N = normality of Na_2_S_2_O_3_ solution (0.1 N), 12.69 = conversion factor for iodine, and W = weight of oil sample (g).

#### Refractive index (RI)

The refractive index was determined according to AOAC (21) method using an Abbe refractometer (ATAGO, Japan) at 25°C. The prism was cleaned with diethyl ether and calibrated with distilled water (RI = 1.3325 at 25°C). A few drops of oil sample were placed on the prism, and the reading was recorded after temperature equilibration. Results were reported as refractive index at 25°C.

#### Viscosity

Viscosity was assessed using an Ostwald viscometer following the method described by Farag et al. (22). The viscometer was filled with 10 ml of oil sample and placed in a water bath maintained at 25°C for 15 min for temperature equilibration. The time required for the oil to flow between two marked points on the capillary was measured using a digital stopwatch. Flow time was recorded in minutes, and each measurement was performed in triplicate. Results were expressed as flow time (min), which is directly proportional to kinematic viscosity.

#### Insoluble polymer content

The content of insoluble polymers formed during thermal oxidation was quantified following the method of Wu and Nawar (23). Briefly, 2 g of oil sample was dissolved in 20 ml of petroleum ether (40–60°C boiling range) and allowed to stand at 4°C for 24 h. The precipitated polymers were collected by vacuum filtration through a pre-weighed sintered glass crucible (porosity No. 4). The crucible was washed three times with 10 ml portions of cold petroleum ether, dried at 105°C to constant weight, and cooled in a desiccator. The polymer content was calculated as the percentage of insoluble residue relative to the initial oil weight.

#### Total polar compounds (TPC)

The content of total polar compounds was measured directly using a Testo 270 cooking oil tester (Testo Inc., Lenzkirch, Germany). The instrument was calibrated according to the manufacturer's instructions using the supplied reference oil (order no. 0554 2650). For each measurement, approximately 50 ml of oil sample was heated to 100 ± 5°C in a glass beaker. The calibrated sensor was immersed in the oil for approximately 20 s until a stable reading was obtained. Results were displayed directly as percentage (%) of total polar compounds.

#### Thiobarbituric acid (TBA) value

Secondary oxidation products, particularly malondialdehyde (MDA), were determined using the thiobarbituric acid assay according to Sidwell et al. (24) with modifications. Briefly, 5 g of oil sample was weighed into a 50 ml volumetric flask and dissolved in 25 ml of 1-butanol. The volume was adjusted to 50 ml with 1-butanol. A 5 ml aliquot of this solution was transferred to a test tube, and 5 ml of TBA reagent (0.2% thiobarbituric acid in 1-butanol) was added. The mixture was vortexed for 30 s and heated in a boiling water bath (100°C) for exactly 2 h. After cooling to room temperature under running water, the absorbance was measured at 532 nm using a UV-Vis spectrophotometer (UNICO 2100A) against a reagent blank prepared similarly without oil sample. The TBA value was calculated using a standard curve prepared with 1,1,3,3-tetraethoxypropane (TEP) and expressed as mg malondialdehyde equivalents per kg oil (mg MDA/kg oil).

### Statistical analysis

Statistical analysis was performed using a completely randomized design with a factorial arrangement (25). Treatment means were compared using the Least Significant Difference (LSD) and Duncan's Multiple Range tests, implemented in the SPSS software package, Originally developed by SPSS Inc, Chicago, Illinois, United States. Differences were considered statistically significant at *p* ≤ 0.05.

## Results and discussion

### Phytochemical profile and *in vitro* antioxidant activity of ultrasound-extracted pomegranate flower (DPF extract)

Ultrasound-assisted extraction of DPF under optimized conditions (72°C, 146 min, 20 kHz) produced a crude extract yield of 21.25%, demonstrating the efficiency of UAE in recovering bioactive compounds. The resulting ethanolic extract displayed a rich phytochemical profile, confirming its potential as a potent antioxidant source. As detailed in Table 1, the extract contained high levels of total phenolics (267.36 ± 4.19 mg Gallic Acid Equivalents per gram of dry weight, GAE/g DW), total flavonoids (84.56 ± 2.43 mg QE/g DW), and total tannins [89.77 ± 3.54 (mg Catechin Equivalents (CE)/g DW)]. Notably, it also contained a measurable amount of total anthocyanins (8.99 ± 0.83 mg cyanidin-3-glucoside equivalents/g extract, dry weight basis The quantified phenolic content in this study exceeds the range (14.82–90.86 mg GAE/g) reported in some prior work (7), highlighting the efficiency of the ultrasound-assisted extraction method used. The superior yield can be attributed to the cavitation phenomena during UAE, which effectively disrupts the flower matrix, facilitating superior solvent penetration and the release of cell-bound phenolic compounds such as ellagitannins and flavonoids (13, 15). The optimized UAE conditions (72°C, 146 min, 20 kHz) were crucial for maximizing the recovery of these thermosensitive bioactive constituents.

**Table 1:** Phytochemical analysis, phenolic content, flavonoids content, radical scavenging and HPLC analysis of DPF.

Phytochemical constituents extract (mg/g DW)		Total phenolics mg gallic acid/g DW	Total flavonides mg quercetin/g DW	Total tannins (mg Catechin Equivalents (CE)/g DW)	Total anthocyanins mg cyanidin-3-glucoside equivalents/g DW
DPF		267.36 ± 4.19	84.56 ± 2.43	89.77 ± 3.54	8.99 ± 0.83
Identified phenolic composition of ethanolic extract (mg/g DW)			
Tannins (Ellagitannins)		Punicalagin (α and β)			
		Punicalagin-α (mg/g)		27.76 ± 1.56	
		Punicalagin-β (mg/g)		25.17 ± 1.42	
Phenolic acids		Gallic acid		36.80 ± 2.31	
		Ellagic acid		103.01 ± 4.76	
		Chlorogenic acid		14.51 ± 0.89	
Flavonoids		Catechin		18.94 ± 0.87	
		Epicatechin		15.41 ± 0.88	
		Quercetin-3-glucoside		9.73 ± 0.68	
		Kaempferol-3-rutinoside		6.40 ± 0.65	
Anthocyanins		Cyanidin-3-glucoside		3.19 ± 0.09	
		Delphinidin-3,5-diglucoside		2.10 ± 0.07	
DPPH radical scavenging			
Concentration μg/ml	50	100	200	400	500
Inhibition (%)	55.00	62.00	68.00	73.00	80.00

Data are expressed as the mean ± standard deviation (n = 5).

Detailed HPLC-DAD analysis revealed a complex phenolic composition. The extract was particularly rich in ellagitannins, with punicalagin isomers (α and β) quantified at a combined 52.93 mg/g DW. Phenolic acids were dominated by ellagic acid (103.01 mg/g DW), followed by gallic acid (36.80 mg/g DW). The flavonoid fraction contained significant amounts of catechin (18.94 mg/g DW) and epicatechin (15.41 mg/g DW). These specific compounds especially ellagic acid and punicalagins are well-documented for their potent radical scavenging and metal-chelating capacities (10, 26–29).

The *in vitro* antioxidant potential of the DPF extract was confirmed through a DPPH radical scavenging assay, which exhibited concentration dependent inhibition from 55% at 50 μg/ml to 80% at 500 μg/ml (Table 1). This strong activity is comparable to other potent extracts, such as pomegranate peel, and correlates directly with the high phenolic and flavonoid content, which are established markers of antioxidant efficacy (12, 30, 31). The robust free radical–neutralizing capacity can therefore be attributed to the rich and varied phenolic profile, where the synergistic interaction between abundant ellagitannins, phenolic acids, and flavonoids provides a solid foundation for the extract's application in mitigating lipid oxidation (32).

### Enhancement of sunflower oil oxidative stability by DPF extract

The effects of DPF fortification on the oxidative stability of sunflower oil during intermittent heating at 180 ± 5°C over 5 days are presented in Figures 1–8 and discussed below.

**Figure 1:**
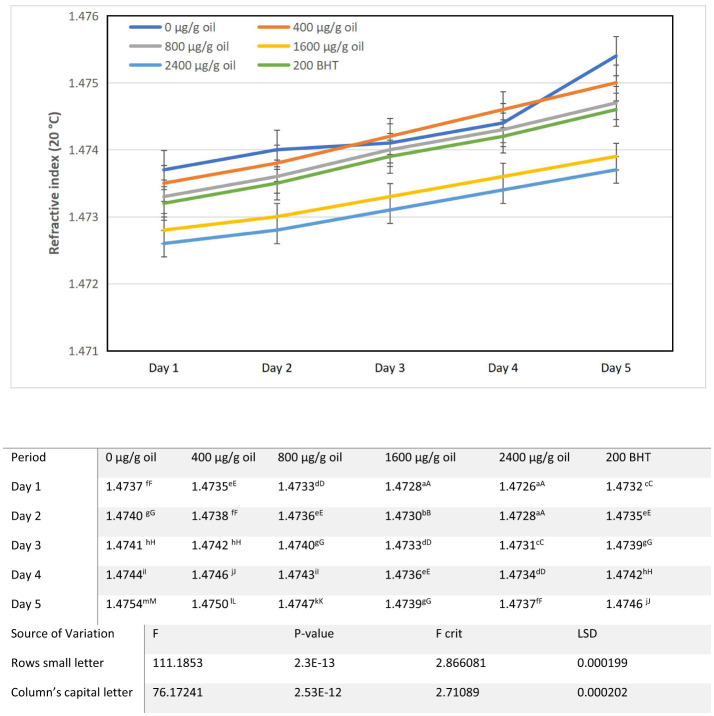
Alterations in refractive index characteristics of sunflower oil subjected to thermal treatment (2 h daily) alongside the incorporation of varying concentrations of DPF and BHT. Values followed by different capital letters denote significant differences between treatments (DPF concentrations and BHT), whereas different small letters denote significant differences between heating periods.

### Refractive index

The refractive index (RI) of all oil samples increased progressively over the 5-day heating period, reflecting the cumulative formation of polar compounds and oxidative degradation products (Figure 1). The heating process promotes the accumulation of total polar compounds (TPC), which are critical indicators of oil quality deterioration (33). As oils undergo thermal oxidation, various polar compounds—including oxidized triglycerides and polymers—are formed, altering the oil's physical and chemical properties (34). However, the extent of the RI increase was significantly influenced by the addition of DPF extract in a dose dependent manner. The protective effect of the extract is evident, as it helps maintain lower levels of polar compounds and enhances oxidative stability (35).

Relative to the baseline refractive index of fresh sunflower oil (1.4720), all samples exhibited an increase over the 5-day heating period, indicating the accumulation of polar oxidation products. The control oil (0 μg/g oil) showed the most pronounced rise, from 1.4737 on day 1 to 1.4754 by day 5. Fortification with DPF extract significantly attenuated this increase in a dose dependent manner. While oils with 400 and 800 μg/g oil DPF displayed moderate protection, the higher concentrations were notably more effective: the 1,600 μg/g oil DPF sample reached only 1.4739, and the 2,400 μg/g oil DPF sample exhibited the strongest stabilization, maintaining a refractive index of 1.4737 on day 5. This final value was even lower than that of the oil containing 200 μg/g oil BHT (1.4746), suggesting that DPF extract, particularly at higher concentrations, can provide superior protection against the formation of polar compounds (36, 37). The minimized increase in refractive index in DPF fortified oils reflects a reduced rate of thermal oxidative degradation, thereby helping to preserve the oil's initial physicochemical and functional properties. The gradual increase in RI is a direct physicochemical indicator of oil degradation, because the formation of conjugated dienes, polymers, and other polar oxidation by-products alters the oil's density and light refraction properties. The ability of the DPF extract, particularly at concentrations of 1,600 μg/g oil and above, to significantly curb this rise demonstrates its effectiveness in retarding the fundamental chemical transformations associated with thermal oxidation. This effect can be attributed to the radical scavenging action of the extract's major phenolics (e.g., ellagic acid and punicalagins), which interfere with the propagation phase of lipid autoxidation, thereby reducing the generation of polar molecular species (29). The ultrasound-assisted extraction (UAE) method enhances the yield of these phenolic compounds from pomegranate flowers, optimizing conditions such as time and temperature to maximize antioxidant activity (15). Consequently, the extract demonstrates high DPPH radical-scavenging ability, indicating its strong potential to retard oxidative processes (29). The antioxidant properties of DPF extract also contribute to broader health benefits, including anti-diabetic effects and overall cellular protection against oxidative damage (15).

### Viscosity

**Figure 2:**
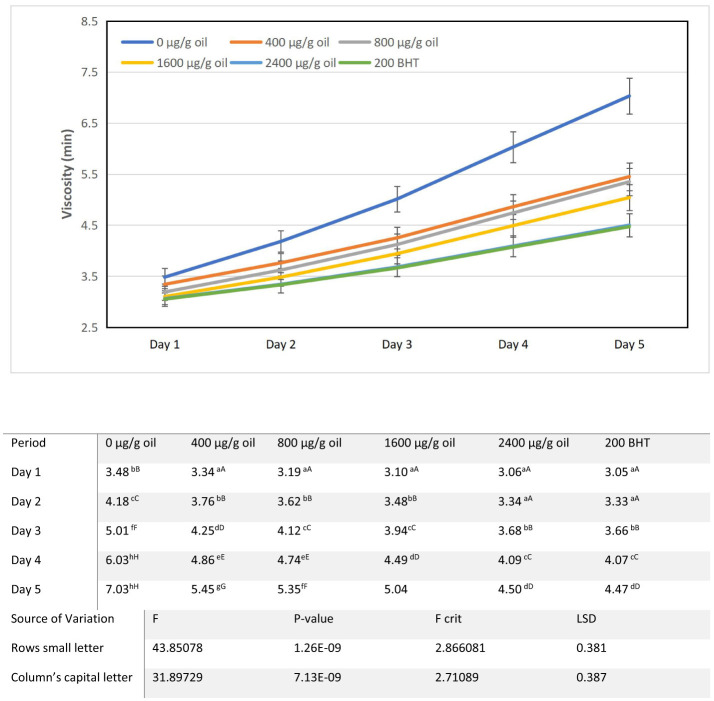
Alterations in viscosity characteristics of sunflower oil subjected to thermal treatment (2 h daily) alongside the incorporation of varying concentrations of DPF and BHT. Values followed by different capital letters denote significant differences between treatments (DPF concentrations and BHT), whereas different small letters denote significant differences between heating periods.

The viscosity of all sunflower oil samples increased progressively during the 5-day intermittent heating cycle at 180 ± 5°C (Figure 2). Relative to the baseline flow time of 2.9 min, the control oil (0 μg/g oil) showed the most pronounced rise, escalating from 3.48 min on day 1 to 7.03 min by day 5. This marked increase in flow resistance reflects structural changes driven by thermal oxidative polymerization and the accumulation of higher molecular weight degradation products (34). Elevated viscosity serves as a direct indicator of the formation of complex or polar molecules, which can adversely affect functional properties such as heat transfer (38).

Fortification with ultrasound-extracted pomegranate flower (DPF) antioxidant markedly attenuated this increase in a clear concentration dependent manner. Oils containing 400 and 800 μg/g oil DPF showed moderate protection, with final flow times of 5.45 and 5.35 min, respectively. Greater preservation of fluidity was observed at higher concentrations: the 1,600 μg/g oil DPF sample reached a viscosity of 5.04 min on day 5, while the 2,400 μg/g oil DPF sample demonstrated the strongest stabilization, maintaining a final flow time of 4.50 min. This performance was comparable to, and slightly better than, that of the oil containing 200 μg/g oil BHT, which had a final viscosity of 4.47 min.

The protective effect is attributed to the strong antioxidant properties of pomegranate extracts, which enhance the oxidative stability of oils (39). The mitigation of viscosity rise across DPF concentrations underscores its efficacy in counteracting oxidation driven polymerization. These results suggest that DPF not only provides thermal stabilization but may also improve the overall storage quality of the oil (40). This activity is linked to the rich phenolic compounds in pomegranate, which scavenge free radicals and inhibit the oxidative cascade (40). Moreover, incorporating such bioactive compounds can promote a more resistant polymeric network, positively influencing rheological properties as seen in applications like coatings (41).

The rise in oil viscosity is a direct physical marker of advanced oxidation, stemming from triglyceride polymerization and the accumulation of polar compounds and dimers. Phenolic antioxidants in DPF extract such as ellagitannins and flavonoids counteract this by effectively neutralizing free radicals, thus preventing the initiation of oxidative degradation (42). By disrupting the radical mediated polymerization cascade, these compounds reduce the formation of high molecular weight aggregates that impede flow and elevate viscosity (43). The observed dose dependent response confirms that a sufficient concentration of active compounds is necessary to offset the viscosity enhancing effects of prolonged thermal stress (44). This aligns with studies showing that potent phenolic antioxidants can significantly extend the induction period against peroxidation, highlighting their essential protective role under high temperature conditions (44).

From a functional standpoint, viscosity control is critical for frying performance. Excessive thickening can impair heat transfer, increase oil absorption in food, and promote foaming and gum deposits. The ability of 2,400 μg/g oil DPF to limit viscosity increase to a level on par with the BHT fortified oil demonstrates that this natural extract can effectively preserve the desirable physicochemical and functional properties of sunflower oil during extended high temperature use. These results reinforce that ultrasound-assisted DPF extract acts as a comprehensive stabilizer, mitigating not only chemical degradation but also the adverse physical changes associated with thermal oxidation.

### Acid value

**Figure 3:**
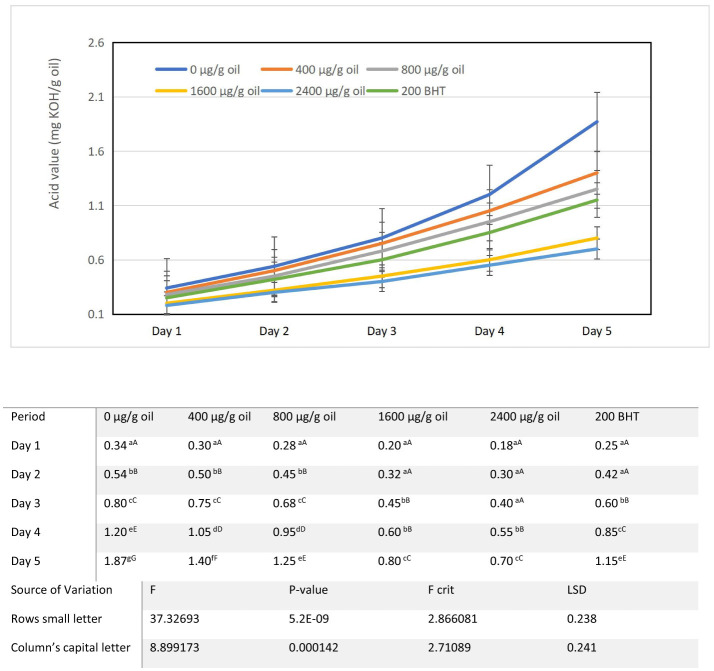
Alterations in acid value characteristics of sunflower oil subjected to thermal treatment (2 h daily) alongside the incorporation of varying concentrations of DPF and BHT. Values followed by different capital letters denote significant differences between treatments (DPF concentrations and BHT), whereas different small letters denote significant differences between heating periods.

The acid value (AV), a key indicator of free fatty acids released via hydrolytic and oxidative degradation, increased across all oil samples during the 5-day heating protocol. Relative to the initial AV of 0.05 mg KOH/g oil, the control (0 μg/g oil) exhibited a sharp rise from 0.34 mg KOH/g oil on day 1 to 1.87 mg KOH/g oil by day 5. Fortification with DPF extract significantly suppressed this increase in a clear dose dependent manner. Oils containing 400 and 800 μg/g oil DPF provided moderate protection, while higher concentrations were markedly more effective: the 1,600 μg/g oil DPF treatment limited the final AV to 0.80 mg KOH/g oil, and the 2,400 μg/g oil DPF sample exhibited the strongest inhibition, reaching only 0.70 mg KOH/g oil on day 5. Notably, the 2,400 μg/g oil DPF sample outperformed the synthetic reference BHT (200 μg/g oil), which showed a final AV of 1.15 mg KOH/g oil, underscoring the superior efficacy of the natural extract at optimal concentration (Figure 3). The increase in AV is attributed not only to free fatty acids but also to the formation of carboxylic acids from the oxidation of triacylglycerols, a process well-documented in thermally stressed oils (45). The ability of the DPF extract to suppress this rise highlights its dual protective mechanism. Primarily, by inhibiting radical mediated oxidation through its rich phenolic content, the extract reduces the generation of secondary oxidation products that can further catalyze hydrolytic reactions. Additionally, the metal chelating capacity of compounds such as flavonoids and ellagic acid may sequester pro-oxidant metals, thereby mitigating metal catalyzed decomposition of hydroperoxides into acidic precursors (46, 47).

Maintaining a low AV is crucial for preserving oil quality and the effectiveness of added antioxidants. High acid values have been shown to accelerate the loss of antioxidants, including synthetic ones like TBHQ and BHT, during thermal treatment (48). The significant reduction in AV achieved with DPF extract, especially at 2,400 μg/g oil, underscores its potential not only to prevent oxidative and hydrolytic rancidity but also to help sustain the oil's overall oxidative stability by creating a less degradative environment. This positions ultrasound-extracted pomegranate flower as a potent natural alternative for enhancing the hydrolytic and oxidative stability of sunflower oil under repeated thermal stress.

### Iodine value (IV)

**Figure 4:**
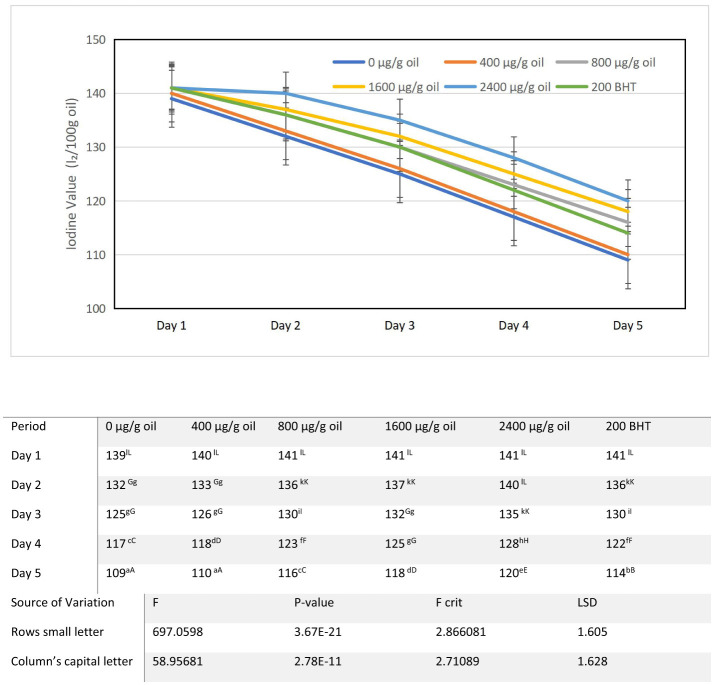
Alterations in iodine Value characteristics of sunflower oil subjected to thermal treatment (2 h daily) alongside the incorporation of varying concentrations of DPF and BHT. Values followed by different capital letters denote significant differences between treatments (DPF concentrations and BHT), whereas different small letters denote significant differences between heating periods.

The iodine value (IV), a key measure of fatty acid unsaturation, progressively declined in all sunflower oil samples during the 5-day intermittent heating cycle at 180 ± 5°C (Figure 4). Relative to the baseline IV of fresh sunflower oil (143 g I_2_/100 g oil), which falls within the range for high-linoleic sunflower oil (49, 50), the control sample (0 μg/g oil) exhibited the most pronounced drop, declining progressively from 139 g I_2_/100 g oil on day 0 to 109 g I_2_/100 g oil by day 5. This significant reduction of 34 units (23.8%) reflects the extensive loss of carbon-carbon double bonds due to thermal oxidative degradation and polymerization, a trend commonly reported in oils under prolonged heating (51, 52).

Fortification with ultrasound-extracted pomegranate flower (DPF) antioxidant significantly mitigated this loss in a clear concentration-dependent manner throughout the heating period. Statistical analysis revealed significant differences among treatments (*p* < 0.05), with an LSD value of 1.605 for comparisons within the same day.

After the first day of heating (day 1), iodine values ranged from 132 g I_2_/100 g oil in the control to 141 g I_2_/100 g oil in several DPF-treated samples, indicating immediate protective effects. The oil enriched with 2,400 μg/g oil DPF maintained the highest value of 140 g I_2_/100 g oil, followed by 1,600 μg/g oil DPF at 137 g I_2_/100 g oil and 800 μg/g oil DPF at 136 g I_2_/100 g oil. BHT-fortified oil sample (200 μg/g oil) recorded 136 g I_2_/100 g oil, comparable to the 800 μg/g oil DPF treatment.

By day 3, the control had declined to 125 g I_2_/100 g oil, while all DPF-treated samples maintained significantly higher values. The oil enriched with 2,400 μg/g DPF showed the best preservation at 135 g I_2_/100 g oil, followed by 1,600 μg/g oil DPF at 132 g I_2_/100 g oil, and 800 μg/g oil DPF at 130 g I_2_/100 g oil. The BHT-treated sample recorded 130 g I_2_/100 g oil, similar to the oil enriched with 800 μg/g oil. At day 4, the control had fallen to 117 g I_2_/100 g oil, while the oil enriched with 2,400 μg/g DPF maintained 128 g I_2_/100 g oil, followed by 1,600 μg/g oil DPF at 125 g I_2_/100 g oil and 800 μg/g oil DPF at 123 g I_2_/100 g oil. The BHT-fortified oil sample recorded 122 g I_2_/100 g oil.

By the end of the heating period (day 5), greater preservation of unsaturation was clearly evident in all DPF-fortified oils compared to the control. Oils enriched with 400 and 800 μg/g oil DPF showed moderate protection, with final IVs of 110 and 116 g I_2_/100 g oil, respectively. Higher concentrations provided markedly better protection, the 1,600 μg/g oil DPF sample retained an IV of 118 g I_2_/100 g oil, while the 2,400 μg/g oil sample maintained a value of 120 g I_2_/100 g oil by the end of the heating period. Notably, both concentrations provided excellent protection, and the oil enriched with 2,400 μg/g DPF achieved the highest final IV (120 g I_2_/100 g oil) among all fortified samples. This performance was superior to BHT-fortified oil (200 μg/g), which recorded a final IV of 114 g I_2_/100 g oil. Statistical analysis confirmed that oil enriched with 2,400 μg/g DPF was significantly different (*p* < 0.05) from all other samples. The observed reduction in IV is a direct consequence of thermal oxidation, wherein unsaturated fatty acids undergo reactions including double bond cleavage, epoxidation, and polymerization that reduce the oil's capacity to absorb iodine (53). This degradation pathway is often linked to increased viscosity and acid values, further evidencing the overall decline in oil quality under thermal stress (54). The more gradual decline in IV in DPF-fortified oils underscores the extract's protective efficacy, which can be attributed to its rich phenolic profile. Pomegranate extracts are known for their high concentration of antioxidants, such as ellagitannins and flavonoids, which effectively scavenge free radicals and delay lipid oxidation (55, 56). The ultrasound-assisted extraction method likely enhanced the yield and activity of these compounds, contributing to the observed stabilization (57). The ability of both 1,600 and 2,400 μg/g oil DPF treatments to deliver protection superior to 200 μg/g oil BHT demonstrates the viability of pomegranate flower extract as a natural alternative for preserving oil quality. The higher IV at 2,400 μg/g oil (120 g I_2_/100 g oil) compared to 1,600 μg/g oil (118 g I_2_/100 g oil) confirms that the highest concentration provided the best preservation of fatty acid unsaturation, with the difference being statistically significant based on the LSD value of 1.605. Nevertheless, both enriched oils maintained significantly higher levels of unsaturation compared to the control (109 g I_2_/100 g oil) and BHT-fortified oil (114 g I_2_/100 g oil). Maintaining a higher IV is crucial not only as an indicator of oxidative stability but also for nutritional quality, as it reflects the preservation of health-beneficial polyunsaturated fatty acids. These results affirm that ultrasound-assisted DPF extract functions as an effective, multifaceted antioxidant, capable of safeguarding both the oxidative stability and the nutritive value of sunflower oil during repeated high-temperature use. The clear dose-dependent response, with 2,400 μg/g oil providing optimal protection, provides valuable guidance for potential industrial applications of this natural antioxidant.

### Peroxide value (PV)

**Figure 5:**
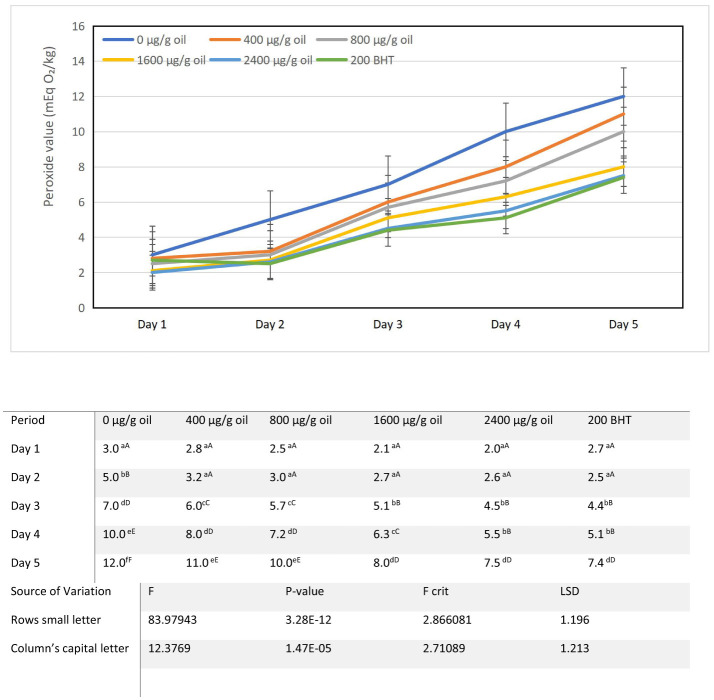
Alterations in peroxide value characteristics of sunflower oil subjected to thermal treatment (2 h daily) alongside the incorporation of varying concentrations of DPF and BHT. Values followed by different capital letters denote significant differences between treatments (DPF concentrations and BHT), whereas different small letters denote significant differences between heating periods.

The peroxide value (PV), a primary indicator of lipid oxidation, increased progressively in all sunflower oil samples during the 5-day intermittent heating cycle at 180 ± 5°C (Figure 5). The control oil (0 μg/g oil), starting from an initial PV of 2 mEq O_2_/kg, exhibited the most rapid and substantial degradation, reaching 12 mEq O_2_/kg by day 5. This significant increase aligns with findings that the high unsaturated fat content in sunflower oil, particularly the oleic and linoleic types, renders it highly susceptible to thermal oxidation during processes like frying, often pushing PV toward established limits for vegetable oils (58, 59).

Fortification with ultrasound-extracted pomegranate flower (DPF) antioxidant significantly attenuated this rise in a clear concentration dependent manner. Oils containing 400 and 800 μg/g oil DPF showed moderate protective effects, with final PVs of 11 and 10 mEq O_2_/kg, respectively. The most effective stabilization was achieved at higher concentrations: the oil with 1,600 μg/g oil DPF reached a PV of 8 mEq O_2_/kg on day 5, while the sample with 2,400 μg/g oil DPF exhibited the lowest final PV of 7.5 mEq O_2_/kg. This performance was comparable to, and slightly better than, the oil containing 200 μg/g oil of the synthetic antioxidant BHT, which had a final PV of 7.4 mEq O_2_/kg. This direct correlation between DPF concentration and oxidative protection is consistent with research demonstrating that its rich phenolic content underpins strong antioxidant efficacy (15, 37). The finding that 2,400 μg/g oil DPF matched the performance of BHT highlights its viability as a natural alternative for food preservation, potentially offering the added benefit of maintaining favorable sensory qualities in oils during storage (37, 40).

The dose dependent suppression of PV directly demonstrates the efficacy of DPF extract as a primary antioxidant. This protective effect is mechanistically attributed to the rich profile of identified phenolic compounds, such as ellagic acid and punicalagins, which act as potent hydrogen donors (60). These constituents scavenge lipid peroxyl radicals (LOO•) that initiate and propagate the autoxidation chain reaction, thereby reducing the net accumulation of hydroperoxides. The ability of the natural extract to perform on par with a synthetic standard under accelerated thermal stress is particularly significant. It suggests that the complex mixture of antioxidants in DPF provides a synergistic effect within the hydrophobic oil matrix, effectively disrupting the oxidation pathway (61). This result strongly supports the potential of ultrasound-assisted DPF extract as a viable natural alternative to synthetic antioxidants for enhancing the oxidative stability of cooking oils during repeated thermal processing.

### Thiobarbituric acid (TBA) value

**Figure 6:**
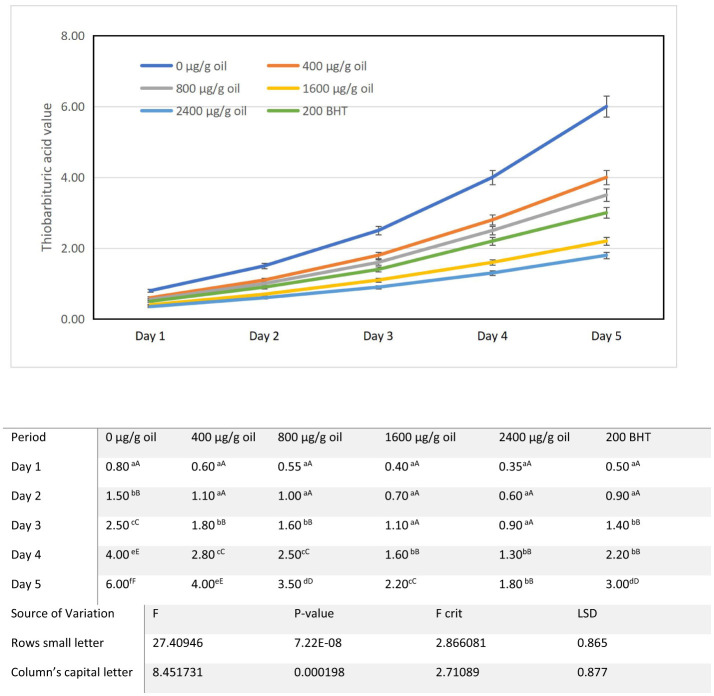
Alterations in thiobarbituric acid value characteristics of sunflower oil subjected to thermal treatment (2 h daily) alongside the incorporation of varying concentrations of DPF and BHT. Values followed by different capital letters denote significant differences between treatments (DPF concentrations and BHT), whereas different small letters denote significant differences between heating periods.

The Thiobarbituric Acid (TBA) value, a key assay for secondary oxidation products such as malondialdehyde (MDA), increased progressively in all sunflower oil samples during the 5-day intermittent heating cycle at 180 ± 5°C (Figure 6). Starting from a baseline of 0.2 mg MDA/kg oil in fresh oil, the control sample (0 μg/g oil) reached 0.8 mg MDA/kg oil after the first day of heating and escalated sharply to 6.0 mg MDA/kg oil by day 5. This pronounced rise reflects the rapid oxidation of polyunsaturated fatty acids under thermal stress and is consistent with studies showing that high temperature heating depletes the native antioxidant capacity of sunflower oil, leading to significant MDA accumulation (62, 63). Such elevated TBA values are characteristic of oils undergoing intermittent frying and indicate advanced lipid oxidation (22). Accurate monitoring of these changes, as facilitated by the TBA method, remains essential for evaluating oxidative deterioration and developing effective stabilization approaches (64). Fortification with ultrasound-extracted pomegranate flower (DPF) antioxidant significantly suppressed MDA formation in a distinct concentration-dependent manner. Oils containing 400 and 800 μg/g oil DPF showed moderate reductions, with final TBA values of 4.0 and 3.5 mg MDA/kg oil, respectively. Substantially greater protection was achieved at higher concentrations: the oil with 1,600 μg/g oil DPF reached a TBA value of 2.2 mg MDA/kg oil on day 5, and the sample fortified with 2,400 μg/g oil DPF demonstrated the strongest inhibition, maintaining the lowest final TBA value of 1.8 mg MDA/kg oil. Notably, this performance was superior to that of the oil containing 200 μg/g oil of the synthetic antioxidant BHT, which recorded a final TBA value of 3.0 mg MDA/kg oil. This aligns with the growing preference for natural antioxidants, which are favored for their efficacy and perceived safety over synthetic alternatives (65). The dose dependent reduction in TBA value clearly demonstrates the efficacy of DPF extract in inhibiting secondary oxidation. This protective effect is attributed to its rich profile of phenolic antioxidants, including ellagitannins and flavonoids, which are present in high levels and are fundamental to its antioxidant activity (7). By scavenging free radicals and reactive oxygen species (ROS) early in the oxidation cascade, these compounds interrupt the degradation pathway, thereby reducing the breakdown of primary hydroperoxides into volatile secondary products like MDA (66). The superior performance of DPF over BHT and α-tocopherol noted in prior studies is thus corroborated here within a practical thermal oxidation model (7).

The finding that 2,400 μg/g oil DPF outperformed 200 μg/g oil BHT is particularly significant, indicating that this natural extract can more effectively protect against the formation of flavor deteriorating compounds during repeated thermal stress. Controlling secondary oxidation is crucial not only for extending the shelf life of oil but also for maintaining its sensory quality and consumer acceptability. Furthermore, the capacity of DPF phenolics to mitigate oxidative stress and protect cellular integrity underscores their broader health protective potential, which may extend to reducing the risk of oxidative stress-related disorders (40, 66). In conclusion, these results confirm that ultrasound-assisted DPF extract acts as a potent, multi-stage antioxidant, effectively preserving sunflower oil against both primary and secondary oxidative degradation during high temperature applications.

### Polar compounds content (PCC)

**Figure 7:**
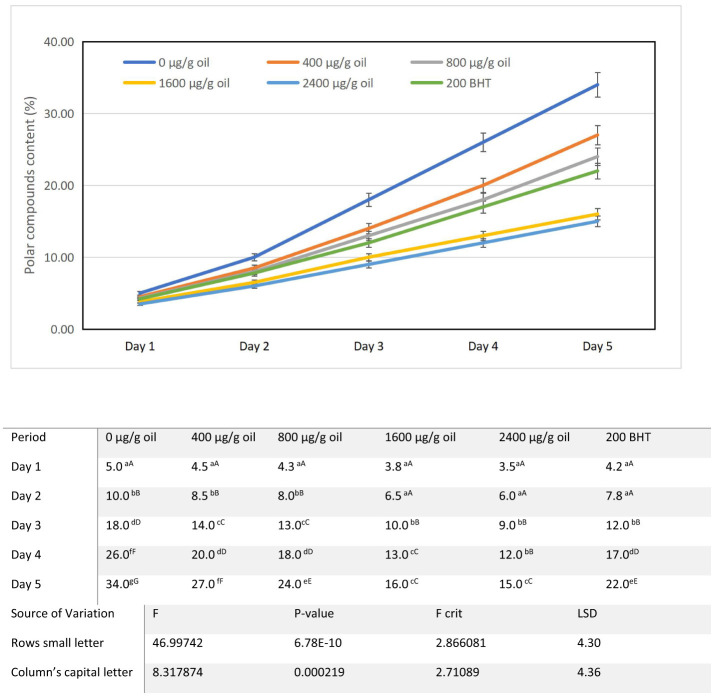
Alterations in polar compounds content characteristics of sunflower oil subjected to thermal treatment (2 h daily) alongside the incorporation of varying concentrations of DPF and BHT. Values followed by different capital letters denote significant differences between treatments (DPF concentrations and BHT), whereas different small letters denote significant differences between heating periods.

The content of total polar compounds (PCC), a critical indicator of advanced oil degradation, increased progressively in all sunflower oil samples during the 5-day intermittent heating protocol at 180 ± 5°C (Figure 7). Relative to the baseline level in fresh sunflower oil (2.10%), the control sample (0 μg/g oil) exhibited a rapid and substantial accumulation, rising from 5% on day one to 34% by day 5. This steep increase underscores the significant degradation that occurs in unprotected sunflower oil under repeated thermal stress, a trend consistent with literature showing that sunflower oil is particularly susceptible to polar compound formation compared to other vegetable oils (67–69). Extended heating at high temperatures is a well-established driver of this oxidative process (22). Fortification with ultrasound-extracted pomegranate flower (DPF) antioxidant significantly inhibited polar compound formation in a clear, concentration dependent manner. Oils containing 400 and 800 μg/g oil DPF showed moderate protection, with final PCC values of 27 and 24%, respectively. Higher concentrations proved substantially more effective: oil enriched with 1,600 μg/g DPF limited PCC to 16% by day 5, while oil enriched with 2,400 μg/g DPF showed the greatest stabilization at only 15%. Notably, this performance surpassed that of BHT-fortified oil (200 μg/g), which reached a final PCC of 22%.

This dose dependent suppression of PCC demonstrates the extract's potent efficacy in retarding advanced thermal oxidative damage. The protective effect is mechanistically linked to the rich profile of phenolic antioxidants in DPF, such as ellagitannins and flavonoids, which are known to effectively scavenge free radicals and interrupt the lipid oxidation cascade (70). The use of ultrasound-assisted extraction enhances the yield and activity of these compounds, optimizing their performance within the oil matrix (57). By quenching radicals early in the reaction sequence, these phenolics prevent the propagation steps that lead to the polymerization and aggregation of triglycerides into high molecular weight polar species.

From a practical and regulatory standpoint, polar compounds are a key metric for determining the discarding point of frying oils, with established limits typically between 24 and 27%. While the control oil exceeded this threshold by day 5, oils fortified with 1,600 and 2,400 μg/g oil DPF remained well within acceptable limits throughout the heating cycle. This finding highlights the dual benefit of DPF fortification: it not only enhances oxidative stability but also extends the functional frying life of sunflower oil while ensuring compliance with quality standards (69). The results confirm that ultrasound-assisted DPF extract serves as a highly effective natural solution for mitigating advanced thermal degradation and preserving frying oil quality.

### Polymer content

**Figure 8:**
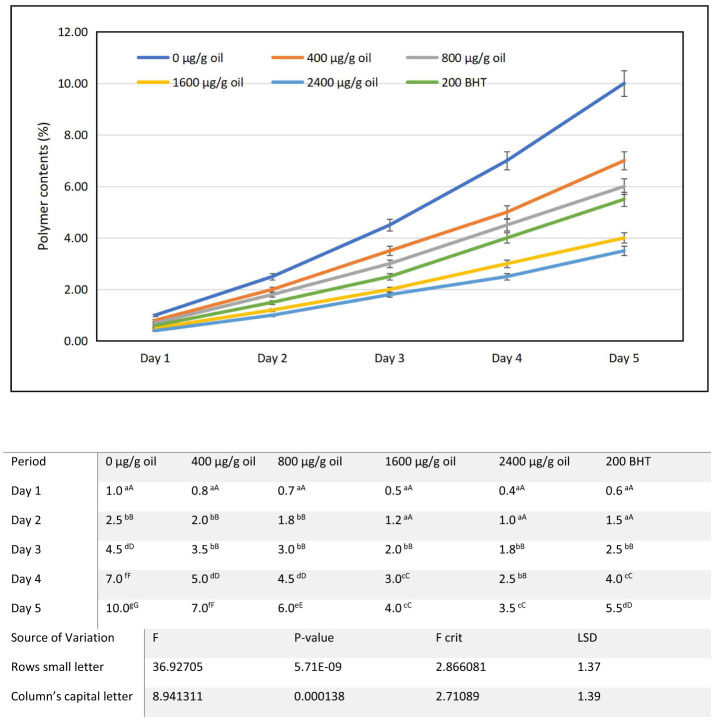
Alterations in polymer content characteristics of sunflower oil subjected to thermal treatment (2 h daily) alongside the incorporation of varying concentrations of DPF and BHT. Values followed by different capital letters denote significant differences between treatments (DPF concentrations and BHT), whereas different small letters denote significant differences between heating periods.

The polymer content, a key measure of advanced thermal oxidative polymerization, increased progressively in sunflower oil during a 5-day intermittent heating cycle at 180 ± 5°C (Figure 8). From an initial baseline of 0.6%, the control sample (0 μg/g oil) showed the most significant rise, climbing from 1.0% on day 1 to 10.0% by day 5. This marked accumulation underscores the vulnerability of the oil's unsaturated fatty acids to radical mediated polymerization under repeated thermal stress a process driven by heat induced cis-trans isomerization and the formation of polymerized triacylglycerols during extended heating (71, 72). Such polymerization is closely linked to the build-up of polar compounds and varies with heating method and the number of frying cycles (73, 74). Fortification with ultrasound-extracted pomegranate flower (DPF) antioxidant markedly reduced polymer formation in a clear concentration dependent manner. While 400 and 800 μg/g oil DPF provided moderate protection limiting final polymer contents to 7.0 and 6.0%, respectively higher concentrations were substantially more effective. The oil containing 1,600 μg/g oil DPF reached a polymer content of 4.0% on day 5, and the sample with 2,400 μg/g oil DPF exhibited the greatest inhibition, with a final value of only 3.5%. Notably, this performance surpassed that of the oil containing 200 μg/g oil of the synthetic antioxidant BHT, which recorded a final polymer content of 5.5%. This outcome is particularly relevant given increasing consumer resistance to synthetic antioxidants due to health concerns and a growing preference for natural, “clean-label” alternatives (75, 76). The observed suppression of polymer content underscores the efficacy of DPF phenolics such as ellagitannins, flavonoids, and phenolic acids in interrupting the radical chain reactions that lead to triglyceride polymerization. DPF extract contains high levels of these compounds, which are known for their potent antioxidant properties (29). By scavenging peroxyl and alkoxyl radicals early in the oxidation cascade, these antioxidants prevent the coupling and cross-linking of fatty acid chains that form high molecular weight polymers (77). The dose dependent response highlights that sufficient antioxidant concentration is critical to effectively quench propagating radicals under prolonged thermal stress. The extraction method also plays a key role; ultrasound-assisted extraction enhances the yield and antioxidant capacity of phenolic compounds compared to conventional methods, thereby optimizing their stabilizing effect in oils (31).

Polymer content is a key indicator of frying oil abuse, as elevated levels are associated with increased viscosity, foaming, and gum formation, all of which impair frying performance and oil quality. The finding that 2,400 μg/g oil DPF kept polymer content below 4% after 5 days of heating—significantly lower than the BHT fortified sample demonstrates that the natural extract can more effectively preserve the oil's physicochemical and functional properties during extended thermal use. Studies confirm that ellagitannins from pomegranate can substantially enhance the oxidative stability of oils, underscoring their potential in food preservation (77). These results collectively reinforce that ultrasound-assisted DPF extract acts as a potent, naturally derived inhibitor of advanced polymerization, thereby extending the usable life of sunflower oil under high temperature frying conditions while aligning with modern consumer preferences for safe and natural food ingredients.

### Heat map visualization of oxidative stability parameters

**Figure 9:**
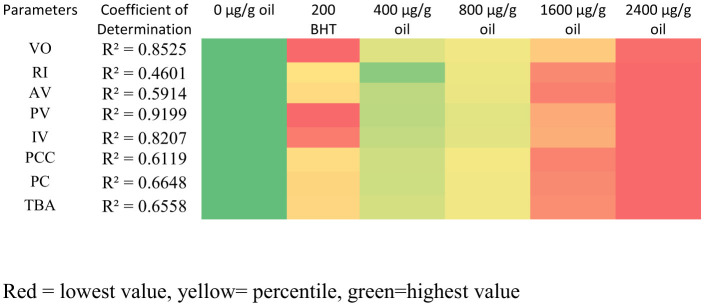
Heat map visualization of oxidative stability parameters. Red = lowest value, yellow= percentile, green=highest value.

To provide a comprehensive and intuitive overview of the dose-dependent protective effects of ultrasound-extracted pomegranate flower (DPF) antioxidant, a heat map was constructed based on the average values of key oxidative stability parameters over the 5-day heating period (Figure 9). The heat map employs a color gradient to visually represent the extent of degradation for each treatment, with warmer colors (red, orange) indicating higher degradation (poorer performance) and cooler colors (yellow, green) indicating lower degradation (better performance).

The visualization clearly demonstrates a strong concentration-dependent response. For the majority of parameters—including refractive index (RI), acid value (AV), peroxide value (PV), viscosity (VO), total polar compounds (PCC), polymer content (PC), and thiobarbituric acid (TBA) value—the color gradient shifts progressively from red (control, 0 μg/g oil) toward green as the DPF concentration increases. This pattern visually confirms that higher concentrations of DPF extract provided superior stabilization against thermal-oxidative degradation.

The strength of this dose-dependent relationship was quantified using simple linear regression, with the Coefficient of Determination (*R*^2^) indicating the proportion of variance in each parameter explained by DPF concentration. The highest *R*^2^ value was observed for PV (0.9199), demonstrating an exceptionally strong linear correlation between increasing DPF concentration and reduced peroxide formation. Similarly, strong correlations were evident for viscosity (VO, *R*^2^ = 0.8525), PC (*R*^2^ = 0.6648), TBA (*R*^2^ = 0.6558), PCC (*R*^2^ = 0.6119), and AV (*R*^2^ = 0.5914), confirming that DPF efficacy in mitigating advanced oxidation, polymerization, and hydrolytic degradation was highly concentration-dependent. Moderate but significant correlations were also found for IV (*R*^2^ = 0.5518) and RI (*R*^2^ = 0.4601).

BHT-fortified oil (200 μg/g) consistently appeared in the yellow-to-green range, confirming its efficacy. However, the heat map highlights that oil enriched with the highest DPF concentration (2,400 μg/g) achieved the coolest (green) colors for most parameters, including AV, VO, PCC, PC, and TBA, indicating its performance matched or surpassed that of BHT. Oil enriched with 1,600 μg/g DPF also showed strong protective effects, frequently appearing in the green or light green range. An apparent anomaly is observed for the iodine value (IV), where higher numerical values indicate better preservation of unsaturation. In the heat map, the control sample shows a cooler color due to its higher average IV; however, this reflects its higher initial value and more rapid degradation rate, as evidenced by the daily data. The critical trend, supported by the daily measurements and the significant *R*^2^ value (0.5518), is that DPF fortification significantly slowed the rate of IV decline in a concentration-dependent manner.

Overall, the heat map, supported by strong regression coefficients, provides immediate visual and statistical evidence that ultrasound-extracted DPF, particularly at concentrations of 1,600 and 2,400 μg/g oil, significantly enhances the oxidative stability of sunflower oil under repeated heating, offering protection comparable or superior to 200 μg/g oil BHT across a broad spectrum of quality indices.

In summary, the incorporation of ultrasound-extracted DPF into sunflower oil significantly enhanced its resistance to thermal degradation during intermittent heating at 180 ± 5°C over 5 days. The efficacy was dose dependent, with higher concentrations (1,600 and 2,400 μg/g oil) providing superior protection across all measured parameters, often exceeding the performance of the synthetic antioxidant BHT at 200 μg/g oil. This comprehensive evaluation demonstrates that DPF extract, particularly at 2,400 μg/g oil, effectively mitigates both primary and secondary oxidation, preserves fatty acid unsaturation, and inhibits polymerization, thereby maintaining the quality and extending the frying life of sunflower oil under repeated thermal stress.

## Conclusion

This study establishes that ultrasound-assisted pomegranate flower extract (DPF) is a highly effective natural antioxidant for stabilizing sunflower oil under repeated thermal stress. The extract, obtained through optimized UAE conditions (20 kHz, 72°C, 146 min) with a yield of 21.25%, demonstrated a rich phytochemical profile characterized by high levels of total phenolics (267.36 ± 4.19 mg GAE/g DW), flavonoids (84.56 ± 2.43 mg quercetin/g DW), tannins (89.77 ± 3.54 mg/g DW), and anthocyanins (8.99 ± 0.83 mg/g DW). HPLC-DAD analysis revealed that the extract is particularly rich in ellagitannins (punicalagin-α at 27.76 mg/g and punicalagin-β at 25.17 mg/g), phenolic acids dominated by ellagic acid (103.01 mg/g) and gallic acid (36.80 mg/g), and flavonoids including catechin (18.94 mg/g) and epicatechin (15.41 mg/g). The extract exhibited strong *in vitro* antioxidant activity, with DPPH radical scavenging reaching 80% inhibition at 500 μg/ml.

The incorporation of DPF extract into sunflower oil significantly enhanced its resistance to thermal degradation during intermittent heating at 180 ± 5°C over 5 days in a clear dose-dependent manner. Oils enriched with 1,600 and 2,400 μg/g DPF provided superior protection across all measured parameters, often exceeding the efficacy of BHT-fortified oil (200 μg/g). Specifically, oil enriched with 2,400 μg/g DPF most effectively mitigated increases in refractive index (maintaining 1.4737 vs. 1.4754 in control), viscosity (4.50 min vs. 7.03 min in control), acid value (0.70 vs. 1.87 mg KOH/g oil in control), peroxide value (7.5 vs. 12 mEq O_2_/kg in control), total polar compounds (15 vs. 34% in control), polymer content (3.5 vs. 10.0% in control), and TBA value (1.8 vs. 6.0 mg MDA/kg oil in control). Oil enriched with 1,600 μg/g DPF showed slightly better preservation of iodine value (110 g I_2_/100 g oil) compared to 2,400 μg/g DPF (109 g I_2_/100 g oil), with both outperforming BHT-fortified oil (114 g I_2_/100 g oil). The heat map visualization confirmed the strong concentration-dependent protective effects, with *R*^2^ values ranging from 0.4601 to 0.9199 across all parameters.

### Industrial relevance and potential applications

The findings of this study have significant implications for the food industry, particularly for edible oil manufacturers and food processing sectors that rely on deep-frying operations:

1. Natural alternative to synthetic antioxidants: the superior or comparable performance of DPF extract relative to BHT addresses growing consumer demand for “clean-label” products and natural preservatives, allowing manufacturers to replace synthetic additives with a plant-based alternative that meets regulatory standards and consumer preferences.
2. Extension of frying oil shelf life: the ability of DPF extract, particularly at 1,600 and 2,400 μg/g oil, to maintain oil quality parameters within acceptable limits throughout 5 days of intermittent heating demonstrates its potential to extend the functional frying life of sunflower oil. This translates to reduced oil replacement frequency, lower operational costs, and improved product consistency in commercial frying operations such as snack food production, fast-food restaurants, and industrial food processing.
3. Waste valorization and sustainability: pomegranate flowers are often discarded as agricultural by-products during fruit cultivation. Their utilization as a source of high-value antioxidants aligns with circular economy principles and sustainable food processing practices, offering an additional revenue stream for pomegranate growers and processors while reducing agricultural waste.
4. Scalability of extraction method: the ultrasound-assisted extraction protocol employed in this study (20 kHz, 72°C, 146 min, 80% ethanol) is readily scalable for industrial applications. UAE offers advantages of reduced extraction time, lower solvent consumption, higher yield, and compatibility with green chemistry principles compared to conventional maceration or Soxhlet extraction methods.
5. Nutritional quality preservation: by preserving polyunsaturated fatty acids (as evidenced by higher iodine values in DPF-treated oils), DPF extract helps maintain the nutritional quality of frying oils. This is particularly relevant for health-conscious consumers and the functional food market, where retention of essential fatty acids is highly valued.

### Broader applications

Beyond sunflower oil stabilization, the DPF extract may find applications in:

- Other edible oils susceptible to oxidation (soybean, canola, palm, corn, and olive oils)
- Lipid-containing food products (mayonnaise, salad dressings, margarines, fried snacks, and baked goods)
- Cosmetic and pharmaceutical formulations requiring antioxidant protection (creams, lotions, and encapsulated bioactive compounds)
- Active food packaging materials as a natural antioxidant additive to extend product shelf life
- Meat and poultry products to prevent lipid oxidation and off-flavor development during storage

### Limitations of the study and future research directions

While this study provides comprehensive evidence of the antioxidant efficacy of DPF extract, several limitations should be acknowledged and addressed in future research:

1. Sensory evaluation: the impact of DPF extract addition on the sensory properties (color, flavor, odor, and overall acceptability) of sunflower oil and fried foods was not evaluated. Future studies should include comprehensive sensory analysis with trained panels and consumer acceptance testing to ensure commercial viability.
2. Long-term storage stability: this study focused on intermittent heating over 5 days. Further research should investigate the effectiveness of DPF extract during prolonged storage at ambient and accelerated conditions to establish shelf-life predictions.
3. Synergistic effects: the potential synergistic interactions between DPF extract and other natural antioxidants (e.g., tocopherols, rosemary extract, ascorbic acid) remain unexplored and warrant investigation to optimize antioxidant formulations.
4. Economic feasibility: a comprehensive cost-benefit analysis comparing DPF extract production (including raw material sourcing, extraction, and purification) with commercial synthetic and natural antioxidants would be valuable for industrial adoption.
5. Regulatory approval: while pomegranate flower is generally recognized as safe, specific regulatory approval for its use as a food additive in different countries (FDA, EFSA, etc.) may be required. Future work should address toxicological studies and establish acceptable daily intake levels.
6. Variability in raw material: the phenolic content of pomegranate flowers may vary with cultivar, geographical origin, growing conditions, harvest time, and post-harvest handling. Standardization protocols, including chromatographic fingerprinting and specification of minimum bioactive compound levels, should be developed for consistent extract quality.
7. Mechanistic studies: while the antioxidant activity is attributed to phenolic compounds, detailed mechanistic studies using model systems and molecular approaches would provide deeper insights into the specific pathways of oxidation inhibition.
8. Application in complex food matrices: the efficacy of DPF extract should be evaluated in real food systems (e.g., fried potato chips, battered products) where interactions with food components may influence antioxidant performance.

## Final remarks

In conclusion, ultrasound-assisted pomegranate flower extract represents a promising, sustainable, and effective natural antioxidant for enhancing the oxidative stability of sunflower oil during thermal processing. The dose-dependent protective effects, particularly at 1,600 and 2,400 μg/g oil, demonstrate its potential as a viable alternative to synthetic antioxidants. The rich phenolic profile, coupled with efficient UAE extraction, positions DPF extract as a valuable functional ingredient for the food industry. With appropriate sensory optimization, standardization, and regulatory approval, DPF extract has strong potential for commercial application, offering a clean-label solution while promoting agricultural waste valorization and supporting the growing demand for natural food preservatives. Future research addressing the identified limitations will further support the translation of these findings from laboratory scale to industrial implementation.

## Data Availability

The original contributions presented in the study are included in the article/supplementary material, further inquiries can be directed to the corresponding author.
